# An Analysis of the Association Between National Football League Players Association (NFLPA) Ratings and Player Injuries

**DOI:** 10.7759/cureus.110909

**Published:** 2026-06-15

**Authors:** Robert Davidson, Sean Gordon, Abraham Hussain, Scarlett Schneider

**Affiliations:** 1 Orthopaedic Surgery, Augusta University, Medical College of Georgia, Athens, USA; 2 Orthopaedics, Mercer University School of Medicine, Savannah, USA; 3 Sports Medicine, Augusta University/University of Georgia Medical Partnership, Athens, USA

**Keywords:** athletic injuries, athletic trainers, football (american), national football league, player ratings, player satisfaction, strength and conditioning

## Abstract

Introduction

Athletic trainers and strength and conditioning coaches are employed in professional football to prevent injuries and minimize the time players are sidelined due to injuries. Evaluating the effectiveness of athletic trainers and strength and conditioning coaches remains challenging due to the lack of standardized, objective metrics that accurately capture their impact on athlete performance and development. This study aims to assess the predictive value of the National Football League Players Association (NFLPA) rankings for evaluating the effectiveness of athletic trainers and strength and conditioning coaches.

Methods

This study employed a cross-sectional ecological design. Data were collected from publicly available databases to quantify the total number of injuries sustained by players on each National Football League (NFL) team, the total number of weeks players missed due to injury on each team, and the overall rankings for each team’s athletic trainers and strength and conditioning coaches in the 2024-2025 NFL regular season. All data were collected, and Spearman's rank correlation coefficients were obtained. For each of the coefficients obtained, significance was determined based on a sample size (n) of 32 and a significance value (𝛂) of 0.05.

Results

Over the course of the 2024-2025 NFL season, there were a total of 1,149 players who missed at least one game due to injury during the regular season. This accounted for a total of 5,811 weeks missed. The league-wide averages for the number of players who missed a game due to injury and cumulative weeks missed due to injury were 36.097 and 183.645, respectively. NFLPA rankings for athletic trainers showed no meaningful correlation with players who missed a game due to injury (ρ = -0.030) and total weeks missed due to injury (ρ = -0.031). NFLPA rankings for strength and conditioning staff were weakly correlated with players who missed a game due to injury (ρ = -0.267) and total weeks missed due to injury (ρ = -0.238).

Conclusion

There was no significant correlation between the NFLPA rankings for athletic trainers or strength and conditioning coaches and the total number of injuries or total number of weeks missed for each team. While this information can be valuable for players to assess how much other players value their team's trainers and strength and conditioning staff, it offers little to no predictive value for the effectiveness of these programs in preventing injuries and minimizing time missed.

## Introduction

In the United States, the National Football League (NFL) is the most-watched sports league, with over 970 billion minutes watched across 284 televised games between 2022 and 2023 [1]. Professional football is a multibillion-dollar-a-year industry, yet the total number of active players in the NFL each year is less than 2,000 [2]. There are a total of 32 NFL teams, and the total number of NFL players in each franchise is trimmed yearly to an active gameday roster of 53 individuals [2]. Given the high value each of these players provides to their respective teams each week, team owners and coaching staff are motivated to keep their players on the field [3]. One of the most important ways a team can keep its players on the field is by preventing injuries [4]. Injuries significantly impact the lives of athletes on and off the field, and the stakes are even higher for professional athletes [5]. The NFL is aware of this, and efforts have been made in recent years to change the rules of the game to make the game safer for the players [6]. The NFL is constantly searching for new ways to protect its players to allow them to stay on the field and remain healthy both during and after their careers [6].

A unique role employed by each of the NFL teams is that of athletic trainers. Athletic trainers play a crucial role in the primary prevention of injuries and health promotion of athletes at both amateur and professional levels, as well as supporting athletes on their road to recovery following an injury [7,8]. Each NFL team has roughly four athletic trainers, which includes one head athletic trainer, one to two associate head athletic trainers, and three assistants [9]. For athletic trainers to be successful, they must follow evidence-based practices and utilize both communication and problem-solving skills to best care for their players [8,10]. It has been demonstrated that athletic trainers can be a valuable yet cost-effective asset that helps both prevent injuries and address the health needs of athletes [8,11]. Additionally, rehabilitation time and performance following an injury have been shown to improve when athletic training interventions are utilized; however, data are sparse on the best ways to evaluate athletic trainers prospectively in their ability to treat their players effectively due to a lack of standardization across the field [8,11].

In addition to utilizing the services of athletic trainers, another way to help prevent injury in high-level athletes is through the utilization of strength and conditioning programs. It has been shown that increasing strength-training volume and intensity decreases the risk of sports-related injury [12]. This is especially true for preventing overuse injury [13]. Additionally, once an athlete is injured, prior strength and conditioning training has been shown to shorten recovery time and help athletes return to their sport sooner [13]. However, some studies have shown that excessive exercise can be a risk factor for developing overuse injury in certain patient populations [14]. Given that strength and conditioning can both increase and decrease injury risk in athletes, teams need to implement strategies that maximize the protective effects of exercise while minimizing its deleterious effects.

One key individual utilized by NFL teams is the strength and conditioning coach. The role of these coaches extends beyond improving the physical abilities of the athletes they train [14]. They are also responsible for communicating with athletic trainers and other members of the coaching staff to both advocate for their players and play a crucial role in the prevention of injuries [14]. These coaches work closely with athletic trainers to help identify players who are at a higher risk of injury. They take steps to modify the athlete’s workload with the hope of preventing an injury from taking them off the field entirely [14]. While these coaches are in a unique position to help prevent injuries in these athletes, it has been shown that strength and conditioning coaches may be hesitant to utilize evidence-based sports science recommendations and are more likely to implement recommendations from peers or mentors [15].

As athletic trainers and strength and conditioning coaches can vary in their methods for helping athletes on their team, it can be challenging to evaluate the quality of these coaches. While this may be less practical at the amateur level, the NFL has attempted to evaluate these programs through a survey program. Every year, the National Football League Players Association (NFLPA) administers an online census survey to the players who were on a roster for a team in the NFL the previous year [14]. The purpose of this survey is to evaluate workplace conditions and the overall perceived quality of the sports medicine staff for each NFL team to both provide insight for potential players who might switch teams and to promote quality improvement of the various teams in the NFL [14]. Each team is provided with a grade that can be compared to the other teams in the league across 11 categories, including data about the teams’ athletic training staff and strength coaches [14].

While the data collected from the NFL players in this survey provides insight into a team's perceived quality of athletic trainers and strength coaches, it does not directly evaluate how well these trainers and coaches implement evidence-based practices or the generalizability of their programs to other teams. Additionally, these data do not explain to team management how effective their athletic trainers and strength and conditioning coaches are at preventing injuries and getting players back to the game at an earlier time. This is especially important to consider for NFL players because injuries lead to lost wages, and it is also vital for teams as injuries may contribute to a loss in the league standings column [14].

While this tool does not directly evaluate the methods implemented by athletic trainers and strength and conditioning coaches, it may still give players an idea of the overall quality of the athletic training and strength and conditioning staff. The purpose of this study is to determine if there is a correlation between a team's ranking in the NFLPA survey and the injuries sustained by players on their respective teams. We hypothesize that a better team ranking in strength and conditioning coaches and athletic training staff will be associated with lower injury rates and less total game time missed, providing valuable insights to players, coaches, team ownership, and fans of the NFL.

## Materials and methods

Study design

This study utilized a cross-sectional ecological correlation design. Data were aggregated at the team level (n = 32). In this design, the unit of analysis is the group (the NFL team), not the individual player. The objective was to investigate the correlation between a team-level exposure (NFLPA staff rankings) and a team-level outcome (aggregate injury statistics). It is critical to note that this design does not allow for inferences to be made at the individual player level.

Data analysis

Injury information on NFL players was obtained using publicly available data (https://profootballreference.com) from the 2024-2025 season. Team rankings for the categories of “Training Staff” and “Strength coaches” were obtained from the publicly available report from the NFLPA (https://NFLPA.com). The rankings are based on anonymous player surveys/questionnaires that reflect the satisfaction of players with the team’s sports medicine personnel. In the rankings provided, a lower numerical rank indicates a better rating/status. For example, a rank of 1 refers to the highest-ranked training staff or strength coaching group. In comparison, a rank of 32 refers to the lowest-ranked training staff or strength coaching group.

Data were entered into a spreadsheet with each row representing an NFL team and columns representing the team’s respective injury values and NFLPA ranking. Data were then analyzed using the Pearson product-moment correlation coefficient as well as the Spearman rank correlation coefficient, with a significance threshold of p = 0.05 to assess for correlation.

Outcome measures

To determine the number of players who missed at least one regular-season game due to injury, we identified all players listed on the injury report during the regular season and excluded those who were listed but did not miss a game. This variable was analyzed as a raw count and was not adjusted for team roster size or turnover.

To determine total weeks missed due to injury, we summed all regular-season injury report entries obtained from Pro-Football-Reference.com, including players designated as probable, questionable, doubtful, out, physically unable to perform, or injured reserve. Entries in which a player appeared on the injury report but participated in the game were excluded from the final count. Total weeks missed due to injury were analyzed as player-weeks lost, defined as injury report entries associated with missed games rather than unique injury events.

## Results

Over the course of the 2024-2025 NFL season, a total of 1,149 players missed a game due to injury during the regular season. This accounted for a total of 5,811 weeks missed. The league-wide averages per team for the number of players who missed a game due to injury and cumulative weeks missed due to injury were 36.097 and 183.645, respectively.

**Figure 1:**
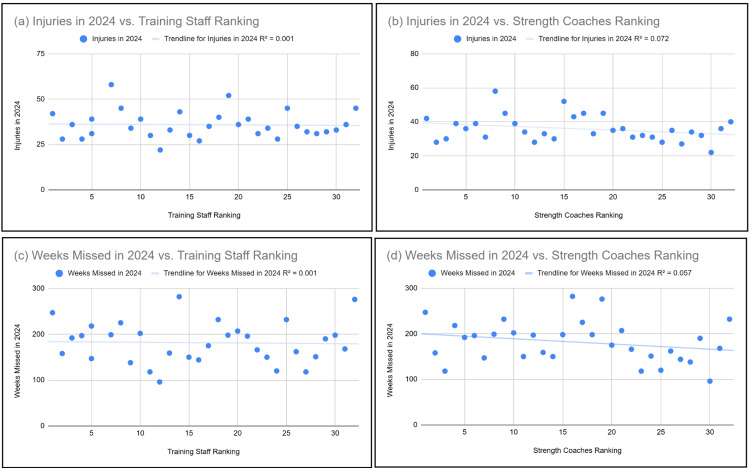
Association between NFL training staff rankings, strength coach rankings, and team injury burden during the 2024 season. Scatterplots demonstrating the association between NFL teams' training staff rankings and strength coach rankings, with injury burden during the 2024 season. Panel (a) shows training staff ranking versus total injuries resulting in at least one missed game (R² = 0.001). Panel (b) shows strength coach ranking versus total injuries resulting in at least one missed game (R² = 0.072). Panel (c) shows training staff ranking versus total injury-related weeks missed (R² = 0.001). Panel (d) shows strength coach ranking versus total injury-related weeks missed (R² = 0.057). Each point represents one NFL team. Linear trendlines are shown to illustrate the overall associations. NFL: National Football League.

Scatterplot analyses demonstrated little to no correlation between team injury burden and either training staff rankings or strength coach rankings (Figure 1). The strongest observed relationship was between strength coach rankings and total injuries, although the association remained weak (R² = 0.072).

T-tests for the correlation coefficients yielded p-values of 0.435 for training staff vs. injuries, 0.063 for strength staff vs. injuries, 0.433 for training staff vs. weeks missed, and 0.089 for strength staff vs. weeks missed. T-tests for Spearman's rank correlation coefficients yielded p-values of 0.574 for training staff vs. injuries, 0.130 for strength staff vs. injuries, 0.420 for training staff vs. weeks missed, and 0.099 for strength staff vs. weeks missed (Tables 1, 2).

**Table 1:** Correlation coefficients for each comparison. Table showing correlation coefficient, t-statistic, and calculated p-value for each of the comparisons examined.

Comparison	Correlation coefficients	t-statistic	p-value
Training staff vs. Injuries	-0.03004287526	-0.164700259	0.4351423326
Strength staff vs Injuries	-0.2674666397	-1.577852142	0.06254368593
Training staff vs. Weeks missed	-0.03099719526	-0.1699419153	0.4330983339
Strength staff vs. Weeks missed	-0.2377500443	-1.380228134	0.08885782924

**Table 2:** Spearman's rank correlation for each comparison. Table showing Spearman's rank correlation coefficient (Spearman’s rho), t-statistic, and calculated p-value for each of the comparisons examined.

Comparison	Spearman's rho value	t-statistic	p-value
Training staff vs. Injuries	0.0344357629	0.1888363675	0.5742536158
Strength staff vs. Injuries	-0.2013929361	-1.149705581	0.1296741987
Training staff vs. Weeks missed	-0.03735879687	-0.2049085444	0.4195137677
Strength staff vs. Weeks missed	-0.2274186466	-1.313559529	0.0994760608

## Discussion

Principal findings

Athletic trainers are first responders to sports injuries and play an active role in the diagnosis, management, and treatment of injury and illness [13]. The quality of these trainers can significantly impact a team's success. Our analysis found no statistically significant correlation between NFLPA ratings of athletic trainers and strength and conditioning coaches and the total number of injuries or weeks missed due to injury. These findings indicate that higher subjective staff ratings were not associated with improved injury outcomes at the team level.

Interpretation of findings

While no association was identified, this finding should be interpreted with caution. Given the ecological study design, which relies on aggregated team-level data, the analysis is inherently limited in its ability to detect individual-level relationships and is susceptible to confounding. As such, the absence of a significant correlation does not necessarily indicate that no true relationship exists.

The subjective nature of player ratings may also contribute to this finding. Players may base evaluations on personal experiences, such as communication style or interpersonal relationships, rather than objective measures of treatment effectiveness. These factors may obscure any underlying association between staff performance and injury outcomes. Future prospective studies focusing on individual-level data, particularly among injury-prone athletes, may help clarify the long-term impact of staff interventions.

When considered in the context of the NFL, these ratings should not be used to infer the effectiveness of training or rehabilitation programs in injury prevention or management. However, they may still provide meaningful insight into player satisfaction and team environment.

Additionally, the use of the term “Training Staff” in the players’ evaluations could introduce errors in the results. While this term was interpreted by the authors of this study to represent athletic trainers, the term “training staff” may be construed by some individuals to include a number of other roles, potentially even strength and conditioning coaches. If some players included strength and conditioning coaches in their evaluation of training staff, this would cause a confounding bias in the comparisons made in this study.

Implications

Athletic trainers and strength and conditioning coaches should prioritize the standardization of training protocols and emphasize evidence-based practices to enhance injury prevention and management. For example, educating athletes and coaches on the limitations of certain protective equipment may reduce preventable injuries [16]. Reducing variability in program design and increasing integration of sports science and continuing education may improve the consistency and effectiveness of injury prevention strategies.

Future research

Future research should focus on developing objective and reliable methods to evaluate athletic trainers and strength and conditioning coaches. Longitudinal studies utilizing individual-level player data will likely provide a more accurate assessment of the relationship between staff performance and injury outcomes. Additionally, further investigation into how staff implement evidence-based recommendations from sports scientists may offer insight into optimizing injury prevention strategies [15].

Limitations

This study has several important limitations, the most critical of which is the potential for ecological fallacy. As our analysis used aggregated team-level data, the lack of correlation at the team level cannot be used to conclude that no relationship exists at the individual player level. For example, within a single team, less injury-prone players may rate staff highly, while more injury-prone players may rate them poorly, masking a true association.

Additionally, the analysis did not control for several important confounding variables that may influence both staff ratings and injury rates, including player age and experience, team playing style, training facilities, team practice and playing surface types, and environmental factors. Injury reports may also have included absences unrelated to injury, such as illness or suspension, further confounding the results.

Another bias that may have influenced the results of this study is the likability bias, with some players ranking more lenient trainers higher than those who are strictly evidence-based in their approach.

Finally, the retrospective nature of the study introduces the potential for selection and recall bias, which may limit the validity of the findings [17].

## Conclusions

There was no significant correlation between the NFLPA's subjective rankings of team athletic trainers or strength and conditioning coaches and the total number of injuries sustained or the total number of weeks players missed due to injury. While this finding is supported by near-zero R² values and non-significant p-values, the interpretation of this result warrants caution. Given the ecological study design, which is inherently limited in its ability to detect individual-level relationships and is susceptible to confounding, the absence of a significant association at the aggregate level does not necessarily indicate that no true relationship exists. Rather, this null finding may reflect methodological constraints, including ecological fallacy and uncontrolled confounders, rather than the impact of these personnel on injury prevention and recovery timelines. Although the NFLPA rankings may still provide value to players in assessing team environment and satisfaction, further research using more robust, individual-level study designs is needed to better evaluate the role of athletic trainers and strength and conditioning coaches in injury prevention and recovery across both professional and amateur sports.
